# Aptamer-mediated hollow MnO_2_ for targeting the delivery of sorafenib

**DOI:** 10.1080/10717544.2022.2149897

**Published:** 2022-12-01

**Authors:** Ziyue Wang, Cuicui Wu, Jinren Liu, Shunxin Hu, Junli Yu, Qiangqiamg Yin, Hongda Tian, Zhipeng Ding, Guiqiang Qi, Li Wang, Liguo Hao

**Affiliations:** a Department of Molecular Imaging, School of Medical Technology, Qiqihar Medical University, Qiqihar, China; b Department of Personnel, the Third Affiliated Hospital of Qiqihar Medical University, Qiqihar, China

**Keywords:** Hollow mesoporous MnO_2_, drug delivery system, HCC, MR imaging

## Abstract

Sorafenib (SRF) presents undesirable effects in clinical treatment, due to the lack of targeting, poor water solubility, and obvious side effects. In this study, we constructed a novel nanodrug carrier system for accurate and efficient delivery of SRF, improving its therapeutic effects and achieving tumor-specific imaging. The hollow mesoporous MnO_2_ (H-MnO_2_) nanoparticles equipped with target substance aptamers (APT) on the surface were used to load SRF for the first time. The resulting H-MnO_2_-SRF-APT could specifically bound to glypican-3 (GPC3) receptors on the surface of hepatocellular carcinoma (HCC), rapidly undergoing subsequent degradation under decreased pH conditions in the tumor microenvironment (TME) and releasing the loaded SRF. In this process, Mn^2+^ ions were used for T_1_-weighted magnetic resonance imaging simultaneously. The *in vitro* cell experiments indicated that H-MnO_2_-SRF-APT showed much more effects on the inhibition in the proliferation of Huh7 and HepG2 HCC cells than that of the non-targeted H-MnO_2_-SRF and free SRF. Besides, the *in vivo* results further confirmed that H-MnO_2_-SRF-APT could effectively inhibit the growth of xenograft tumors Huh7 in the naked mouse with good biosafety. In conclusion, H-MnO_2_-SRF-APT could significantly enhance the therapeutic effect of SRF and is expected to be a new way of diagnosis and treatment of HCC.

## 1. Introduction

Cancer is one of the main killers of human health. Specifically, hepatocellular carcinoma (HCC) is the sixth most commonly diagnosed cancer and leads to the third death of cancer worldwide, killing nearly 800,000 people each year (Sung et al., 2021). However, traditional chemotherapeutic drugs show poor therapeutic efficacy due to the lack of specificity and multi-drug resistance (X. Dong & Mumper, 2010; Mangal et al., 2017). Sorafenib (SRF), a multi-target kinase inhibitor with powerful antitumor and anti-angiogenic effects, has been approved by the US Food and Drug Administration (FDA) as the first-line drug for the treatment of HCC (Semela & Dufour, 2004). SRF can not only inhibit the proliferation of tumors by blocking the RAF/MEK/ERK pathway but also inhibit vascular endothelial growth factor receptor (VEGFR) and platelet-derived growth factor receptor (PDGFR) to block the generation of blood vessels in tumors, representing a dual antitumor effect and significantly improving the survival rate of patients (Kudo et al., 2018; W. Tang et al., 2020). Although SRF exhibited good anti-HCC activity, the adverse reactions such as rashes, diarrhea, and increased blood pressure resulted from nonspecific uptake of SRF by normal tissues, as well as poor water solubility, rapid metabolic clearance, and low absorption efficiency in tumor tissues seriously affected the clinical treatment effect (Mancuso et al., 2011; Xiao et al., 2016; S. Yang et al., 2016; Li et al., 2018).

The nanoparticle drug delivery system (NDDS), consisting of a drug and carrier with a particle size of 3–200 nm (Wathoni et al., 2020), possesses such features as increasing the solubility of drugs, enhancing the stability of drugs in the blood environment, improving the cycle time and targetability of drugs, reducing the toxic side effects of drugs on nontargeted tissues and organs (J. Chen et al., 2021). Previously, versatile nanocarriers such as liposomes (Kroon et al., 2014; J. Liu et al., 2015), solid lipid nanoparticles (H. Zhang et al., 2012; Wang et al., 2018), polymer nanoparticles (Lin et al., 2016; Zheng et al., 2019) and micelles (Pellosi et al., 2016; Su et al., 2018), and mesoporous silica nanoparticles (B. C. Zhang et al., 2020) were developed to deliver SRF, showing significantly enhanced therapeutic efficacy compared to free SRF (F. Chen et al., 2021). However, traditional drug delivery systems exhibit problems such as early drug leakage, low stability, or poor biodegradability (He & Shi, 2011; Huang et al., 2011; Daraee et al., 2016; Mahmoud et al., 2022), resulting in insufficient targeting. Recently developed inorganic material manganese dioxide (MnO_2_) has attracted widespread attention. MnO_2_ nanosystems can react with H^+^ and glutathione (GSH) that substantially exist in tumor microenvironment (TME) (W. Xu et al., 2022). The generating paramagnetic Mn^2+^ significantly increases the contrast of T_1_ magnetic resonance imaging (MRI), which can be used for tumor-specific imaging as well as the development of multifunctional drug carrier systems (X. Xu et al., 2021). In addition, compared with other non-biodegradable inorganic nanomaterials, MnO_2_ nanoparticles can be broken down into harmless and water-soluble Mn^2+^, which can be quickly excreted by renal metabolism without long-term *in vivo* toxicity (W. Zhu et al., 2016; Q. Chen et al., 2018). Meanwhile, MnO_2_ nanoparticles bearing a similar function to peroxidase can catalyze the decomposition of large amounts of H_2_O_2_ in tumors into water and oxygen, thereby alleviating tumor hypoxia and enhancing photodynamic therapy (PDT) (Deng et al., 2022). In addition, MnO_2_ nanostructures can be rapidly degraded and release loaded drugs due to the response to TME. Although various MnO_2_ nanostructures, such as nanoparticles, and nanosheets, have been developed, but usually suffer from low drug loads and inaccurate release of payloads. Hollow nanoparticles with mesoporous structures and large cavities are considered excellent drug carrier systems because of loading large amounts of therapeutic agents. Besides, H-MnO_2_ can be easily functionalized on the surface with targeted ligands and masking agents such as polyethylene glycol (PEG) (G. Yang et al., 2017; Cheng et al., 2020; Ning et al., 2022). In addition, the introduction of targeted ligands enhances the contrast between the imaging target and the background, and increases the concentration of drugs in tumor cells, improving the effectiveness of diagnosis and treatment. Specifically, aptamers (L. Zhu et al., 2021), an oligopoly DNA with specific targeting recognition, can bind to the target with high affinity and specificity, possessing the advantages of low immunogenicity and easily-modified properties, which makes it an ideal approach for the targeting diagnosis and treatment of diseases.

Herein, we developed an unprecedented integrated intelligent platform for HCC diagnosis and treatment based on the H-MnO_2_ carrier system and tumor cell-targeted aptamers. In this study, the surface of H-MnO_2_ was functionalized with AP613-1 aptamers that can be performed as the gatekeeper for controlling SRF release and MRI imaging. As specifically recognized by glypican-3 (GPC3) acceptors that overexpressed in tumor cells (L. Dong et al., 2018; Zhao et al., 2018), AP613-1 aptamers could trigger the degradation of H-MnO_2_-SRF-APT in the TME but are dormant when no GPC3 was encountered. The simultaneously released SRF can accumulate at tumor sites, achieving accurate diagnosis and treatment of HCC. Besides, the *in situ* generated Mn^2+^ ions in TME conditions could enhance T_1_-weighted MRI.

## 2. Materials and methods

### 2.1. Reagents

Aptamer AP613-1 (NH_2_-TAACGCTGACCTTAGCTGCATGGCTTTACATGTTCC-sequence5′-3′) was synthesized by Shanghai Sangon Biotech; tetraethyl orthosilicate (TEOS) was purchased from Aladdin; potassium permanganate (KMnO_4_) was purchased from Henan Yaoye Chemical Products Co., Ltd.; SRF was purchased from Macklin; cetyltrimethylammonium bromide (CTAB) was purchased from Aladdin; polyethylene glycol-2000-*N*-hydroxysuccinimide (PEG-2000-NHS) was purchased from Xi’an Ruixi Biotechnology Co., Ltd.; fluorescein isothiocyanate (FITC) was purchased from Xi’an Ruixi Biotechnology Co., Ltd.; CCK8 kit was purchased in DOJINDO; Hoechst3342 was purchased from Wuhan Service Biotechnology Co., Ltd.; Annexin V-FITC Apoptosis Detection Kit was purchased from Keygen Biotechnology Co., Ltd.; agarose was purchased at Biowest Agarose; 1 × Tris-acetate-EDTA (TAE) buffer was purchased from Solarbio; 6 × RNA/DNA Loading Buffer was purchased from Biomed; The BM5000 DNA Marker was purchased from Biomed.

### 2.2. Synthesis of H-MnO_2_ nanoparticles

Solid silica (sSiO_2_) nanoparticles were synthesized according to previously reported methods (Stöber et al., 1968; F. Tang et al., 2012). The mixture of ethanol (50 mL), deionized water (1 mL), and ammonia water (3.5 mL) was heated to 30 °C, 1.5 mL TEOS was quickly added to the above solution subsequently. After stirring for 2 h, sSiO_2_ was obtained.

To a mixture of sSiO_2_ (50 mg) and H_2_O (8 mL), KMnO_4_ (150 mg) was quickly added, stirred at room temperature for 6 h, followed by centrifugation and washing, and then dispersed in water for later use. Na_2_CO_3_ (220 mg) was added to the above dispersion, stirring for 12 h under 50 °C. After centrifugation, the precipitate was washed with water and ethanol, and dispersed in a solution of methanol (5 mg) and ammonia (0.5 mg), stirring at 70 °C for 48 h. Finally, H-MnO_2_ was obtained by centrifugation and washing.

### 2.3. Synthesis of H-MnO_2_-SRF nanoparticles

To a dispersion of H-MnO_2_ (50 mg) in water (5 mL), a solution of SRF in ethanol was added and stirred overnight at room temperature. After centrifugation, the precipitate was collected. The supernatant was taken to test the content of SRF.

### 2.4. Synthesis of H-MnO_2_-SRF-APT nanoparticles

First, the 10od AP613-1 aptamer was dispersed with PEG-2000-NHS in sterile water (pH = 7 ∼ 8) and stirred overnight. Subsequently, the generated PEG-2000-APT was mixed with H-MnO_2_-SRF and stirred overnight at room temperature to generate H-MnO_2_-SRF-APT.

### 2.5. Characterization of materials

The morphology and elements of the sample were detected by transmission electron microscopy (TEM, JEM2100F). The nanoparticle size and zeta potential were characterized by the Marvin Nanoparticle Size Analyzer (Nano-ZS90). Specific surface area and pore size were measured by the Brunauer–Emmett–Teller (BET) method and Barrett–Joyner–Halenda (BJH) approach (Micromeritics Instrument Corp. ASAP2460). The elemental analysis and valence states of the nanoparticles were detected by the X-ray photoelectron spectrometer (XPS, Thermo ESCALAB 250XI). The crystal structure of the nanoparticles was characterized by the X-ray diffractometer (XRD, BrukerAXS GmbH, D8ADVANCE).

### 2.5. Agarose gel electrophoresis

Dissolved 0.45 g of agarose in 30 mL 1× TAE buffer and heated until all agarose was melted. After cooling, added 2.0 μL of nucleic acid dye and left it for 20–30 min to completely solidify the agarose and placed the agarose in the electrophoresis tank. Pipetted an appropriate amount of H-MnO_2_-SRF-APT, aptamers, and H-MnO_2_-SRF in a centrifuge tube, adding 6× RNA/DNA Loading Buffer to each tube, vortexed all samples well, and then centrifuged instantaneously and sequentially pointed into the agarose gel wells. The setting voltage was 120 V, the current was 100 mA, and the electrophoresis was 30 min. After the end of the electrophoresis, placed the agarose gel into a gel imager for imaging.

### 2.6. Drug load and entrapment rate detection

According to previously reported methods from Liu’s group (X. Liu et al., 2021), SRF powder (10 mg) was dissolved in 20 mL of dimethyl sulfoxide (DMSO) solution to prepare 0.5 mg/mL of SRF solution. Corresponding 250, 100, 50, and 10 μg/mL SRF solutions were obtained through dilution with deionized water. The absorbance values of SRF solution in each concentration were detected under 265 nm UV light to establish the standard curve.

After the SRF completely reacted with H-MnO_2_, the resulting reaction mixture was centrifuged to separate the supernatant and precipitate. The optical density of SRF in the supernatant was determined with an ultraviolet–visible spectrophotometer. The SRF content in H-MnO_2_ was calculated according to the standard curve formula. The drug-loading efficiency (DL) of H-MnO_2_ and encapsulation efficiency (EE) of SRF were also obtained.

$$\mathrm{EE}\;(\%)=\;\frac{\text{mass of drugs loaded in the final carriers}}{\text{mass of drugs fed initially}}\times 100\%$$

$$\mathrm{DL}\;(\%)=\;\frac{\text{mass of drugs loaded in the final carriers}}{\text{mass of the drugs loaded final carriers}}\times 100\%$$

### 2.7. MnO_2_-SRF *in vitro* drug release experiments

PBS buffers of pH = 5.5 (±0.02) and pH = 7.4 (±0.02) were pre-prepared. The two separate dialysis bags (MW = 8.0 to 14 kDa) were added with H-MnO_2_-SRF-APT (5 mL, 1 mg/mL), suspended in 50 mL of PBS buffer (pH = 5.5 and pH = 7.4) respectively, and then placed on a magnetic stirrer at a constant temperature (37 °C) at a speed of 1500 rpm/min. Briefly, 1 mL of H-MnO_2_-SRF-APT solution was removed after 0, 4, 8, 12, 16, 20, and 24 h respectively to determine its absorbance under 265 nm. In this process, the corresponding fresh PBS solution (1 mL) was replenished to the original solution. The SRF release rate was calculated by the following formula:

$$\text{sorafenib release rate }(\%)=\frac{50\times C_{n}+1\times \sum C_{n-1}}{\text{loading amounts of drugs}}\times 100\%$$

*C_n_* represents the concentration (mg/mL) of SRF samples taken at *n* times.

### 2.8. Cell culture

Huh7, HepG2, and human normal hepatocyte line L02 cells were purchased at Fenghui Biotechnology Co., Ltd.; all cells were cultured in Dulbecco’s modified Eagle medium (DMEM) containing 10% fetal bovine serum (FBS) in incubators with 5% CO_2_ under 37 °C for subsequent use.

### 2.9. Cellar uptake experiment

H-MnO_2_-SRF and H-MnO_2_-SRF-APT nanoparticles modified with FITC were incubated with Huh7 and L02 cells and then imaged by laser confocal scanning microscopy (CLSM). The modification of nanoparticles by FITC was performed as follows: took 5 mg of H-MnO_2_-SRF-APT and H-MnO_2_-SRF dispersion solution, added a certain amount of FITC (in sterile water) to H-MnO_2_-SRF-APT and H-MnO_2_-SRF dispersion solution respectively, stirred the mixture at room temperature overnight to give H-MnO_2_-SRF-APT-FITC and H-MnO_2_-SRF-FITC. Huh7 cells and L02 cells with a cell density of 2 × 10^4^/mL in a petri dish were incubated in an incubator for 24 h. FITC-labeled H-MnO_2_-SRF-APT and H-MnO_2_-SRF were added and continued to incubate for another 8 h. Cells were then slowly rinsed three times with PBS (pH = 7.4). The nuclei were labeled with Hoechst, and after 30 min incubated with light avoidance. Finally, the cells were rinsed three times with PBS and added with 1 mL of PBS buffer for imaging by CLSM. And the mean fluorescence intensity was measured by flow cytometry.

### 2.10. Cytotoxicity and proliferation tests *in vitro*

To assess the cytotoxicity of H-MnO_2_ nanocarriers, Huh7 cells were seeded in 96-well plates with the density of 5 × 10^3^/well for 12 h. Next, H-MnO_2_ dispersion in culture medium with different concentrations (0, 5, 10, 25, 50, and 100 μg/mL) was added to the wells. After 24 h, 10 μL of CCK-8 reagent was added to each well. The plates were returned to standard cell incubating conditions for 0.5–1 h (after gentle shaking). After 0.5 h, the OD value at 450 nm was detected by the microplate reader.

Cell viability was calculated using the following formula:

$$\text{cell viability }(\%)=\;\frac{{\mathrm{OD}}_{\mathrm{sample}}\;-\;{\mathrm{OD}}_{\mathrm{blank}}}{{\mathrm{OD}}_{\mathrm{control}}\;-\;{\mathrm{OD}}_{\mathrm{blank}}}\times 100\%$$

In addition, therapeutic effects were evaluated using nanoparticles carrying SRF. In this process, cell viability was tested through CCK-8 assay after free SRF, H-MnO_2_-SRF-APT, and H-MnO_2_-SRF (SRF concentrations were 0, 1, 2, 4, and 8 μg/mL) were incubated with Huh7 and HepG2 cells for 24 h. The methods in determination and data processing were the same as that described in the cytotoxicity tests.

### 2.11. Apoptosis experiments

To evaluate the effect of H-MnO_2_-SRF-APT on apoptosis of liver cancer cells, Huh7 cells with the initial density of 2 × 10^5^/mL were seeded in a 6-well plate. After being cultured for 12 h, each well was added with H-MnO_2_-SRF-APT, H-MnO_2_-SRF, free SRF, and PBS (blank group) and then incubated for another 48 h. The test followed the procedures described in the apoptosis kit. In this study, flow cytometry was used to characterize the cellar apoptosis.

### 2.12. Animal experiments

In this study, the female BALB/C nude mice (4–6 weeks, 16–19 g) were purchased from Dalian Changsheng Biotechnology Co., Ltd., and all animal experiments were performed according to the protocols approved by the Animal Core Facility of Qiqihar Medical University (QMU-AECC-2022-122). All the pre-experimental mice were raised in the SPF environment of Qiqihar Medical University, under a constant and suitable environment with alternating light and dark, as well as temperature (22 ± 1 °C), and humidity (50%–60%) for a period, during which feed and drinking water were adequate. Huh7 cells in the logarithmic growth phase were selected and digested with 0.25% trypsin. After centrifugation, the cells were collected and washed with 4–5 mL of sterile PBS. The concentration of cell suspension was prepared into 2 × 10^6^/mL. Briefly, 100 μL of cell suspension was sucked with a 1 mL insulin needle and inoculated subcutaneously into the right armpit of nude mice. After about 10 days, the size of subcutaneous tumors was grown to about 100 mm^3^, followed by MR imaging of the tumor as well as administration therapy.

### 2.13. Magnetic resonance imaging experiments

To determine the relaxation rate of H-MnO_2_-SRF-APT and evaluate the MR imaging ability of nanoparticles, the concentration of Mn^2+^ was determined to be 130.788 mg/L by Inductively coupled plasma mass spectrometry (ICP-MS). H-MnO_2_-SRF-APT was then dispersed at gradient Mn concentrations (0–1.0 mM) in PBS (pH = 5.5, 7.4) respectively. The longitudinal relaxation rate (*r*_1_) was calculated by linearly fitting the inverse T_1_ relaxation time (1/T_1_) as a function of Mn^2+^ concentration. H-MnO_2_-SRF-APT dispersions were injected into the anesthetized Huh7 tumor-bearing nude mice. The MR images were captured using a 3.0-T clinical MRI scanner before and after the injection at different times (3 h, 5 h, and 7 h).

### 2.14. In vivo tumor treatments and systemic toxicity assessments

Tumor-bearing nude mice were randomly divided into four groups (*n* = 4), and 100 μL of PBS, SRF, H-MnO_2_-SRF, and H-MnO_2_-SRF-APT suspension (SRF = 5 mg/kg) were injected through the tail vein. The first administration was regarded as the first day to record the time, after which it was administered every other day for 15 days. From day one, the tumor’s length and width were measured every other day using a vernier caliper and the body weight of the experimental mice was weighed with an electronic balance. At the end of the treatment cycle, all experimental nude mice were sacrificed, and the tumor and major organs were removed to weigh the quality of the tumor. Subsequently, tumor and organ tissues were paraffin-embedded and sectioned after being fixed in a 4% of paraformaldehyde solution. For histological observation, tumor sections were stained with hematoxylin and eosin (H&E) and then observed by optical microscopy (Leica, Wetzlar, Germany). Calculate the tumor volume according to the formula described below.

Tumor volume calculation formula:

$$V=\frac{a\times b^{2}}{2}$$

where *V* is the tumor volume, *a* is the tumor length, and *b* is the tumor width.

### 2.15. Statistical analysis

Descriptive analyses were presented using mean ± SD for continuous variables. The two-tailed Student’s *t* test was used for data conforming to the normal distribution and the Mann–Whitney’s *U* test was used for data conforming to abnormal distribution in the comparison of the two groups’ data. One-way ANOVA with Tukey’s post-hoc tests was used for comparisons among groups. *p* < .05 was considered statistically significant. Graphical and statistical analysis was done in Origin 2021 and GraphPad Prism 9 version.

## 3. Results and discussion

### 3.1. Characterization

**Figure 1.**
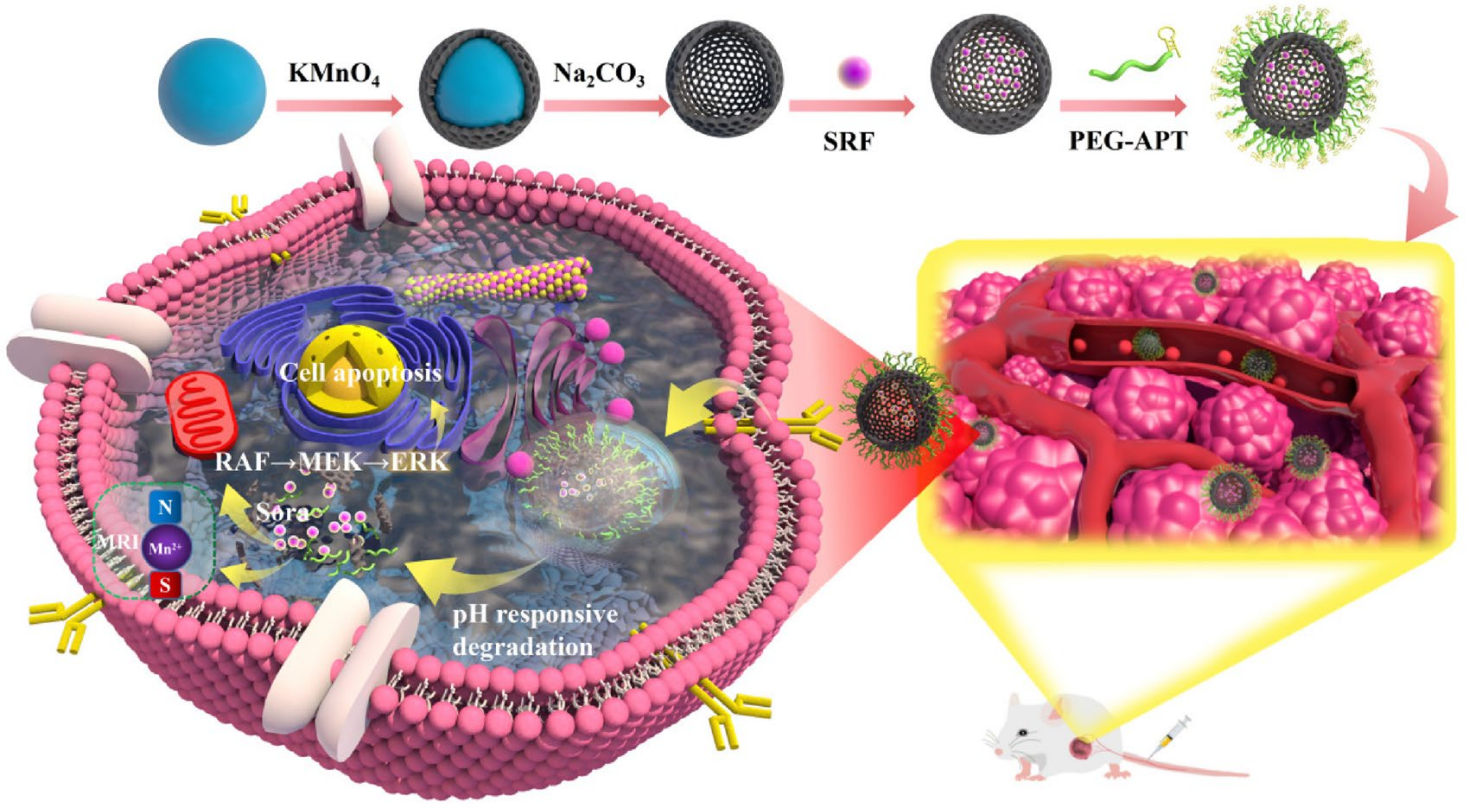
H-MnO_2_-SRF-APT synthesis process and the mechanism of diagnosis and treatment.

**Figure 2.**
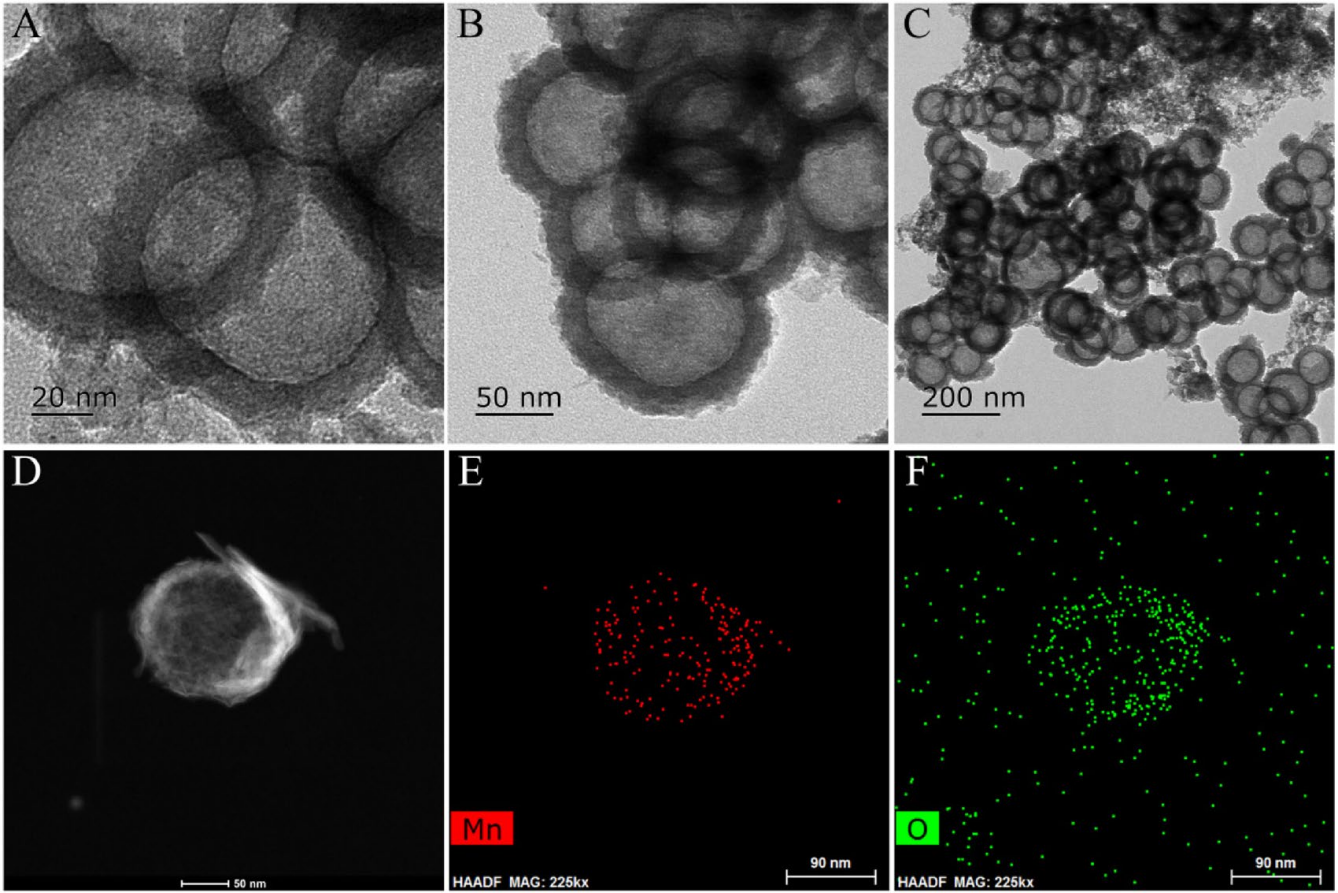
(A–C) TEM images of H-MnO_2_-SRF-APT (D–F) HAADF-STEM image and elemental mapping for H-MnO_2_-SRF-APT.

Synthesis of the H-MnO_2_-SRF-APT as well as the mechanism of diagnosis and treatment is shown in Figure 1. First, manganese dioxide was deposited on the surface of silica nanoparticles (as the hard template). And then the silica template was removed by simple treatment with Na_2_CO_3_ solution to yield the H-MnO_2_ nanocarriers for supporting SRF. Finally, the surface of H-MnO_2_-SRF was modified by PEG-APT to obtain H-MnO_2_-SRF-APT, thereby improving the targeting and water solubility of nanoparticles. H-MnO_2_-SRF-APT presents a uniform hollow spherical form with an average diameter of 100 ± 10 nm through TEM (Figure 2(A–C)). In addition, the element mapping of high-angle annular dark-field scanning (HAADF-STEM) (Figure 2(D–F)) further demonstrates the hollow structure of the prepared H-MnO_2_, and the manganese and oxygen elements contained in the nanoparticles could be distinguished.

**Figure 3.**
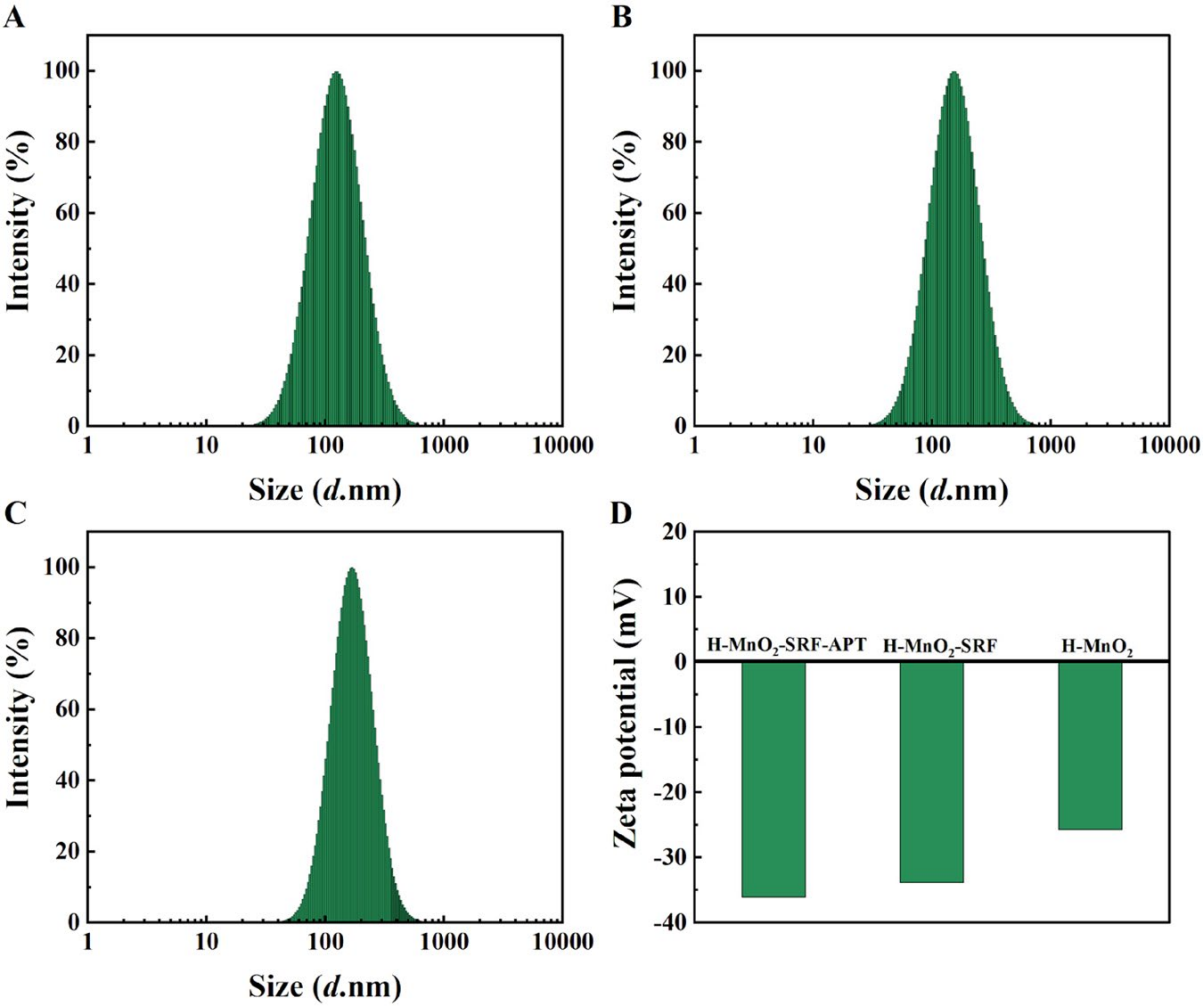
(A–C) Hydrated particle size of H-MnO_2_, H-MnO_2_-SRF, H-MnO_2_-SRF-APT. (D) Potential of different nanoparticles.

The potentials and hydration particle size are shown in Figure 3. In the process of preparing the H-MnO_2_-SRF-APT nanocarrier, the hydration particle size of the particles gradually increased from 130 nm to 160 nm after loading drugs and coating with PEG-APT. After being modified by PEG-APT, the potential of the nanocarrier has been changed from –33.84 mV to –36.06 mV, indicating that the nanoparticles were successfully modified by a negatively charged nucleic acid aptamer.

**Figure 4.**
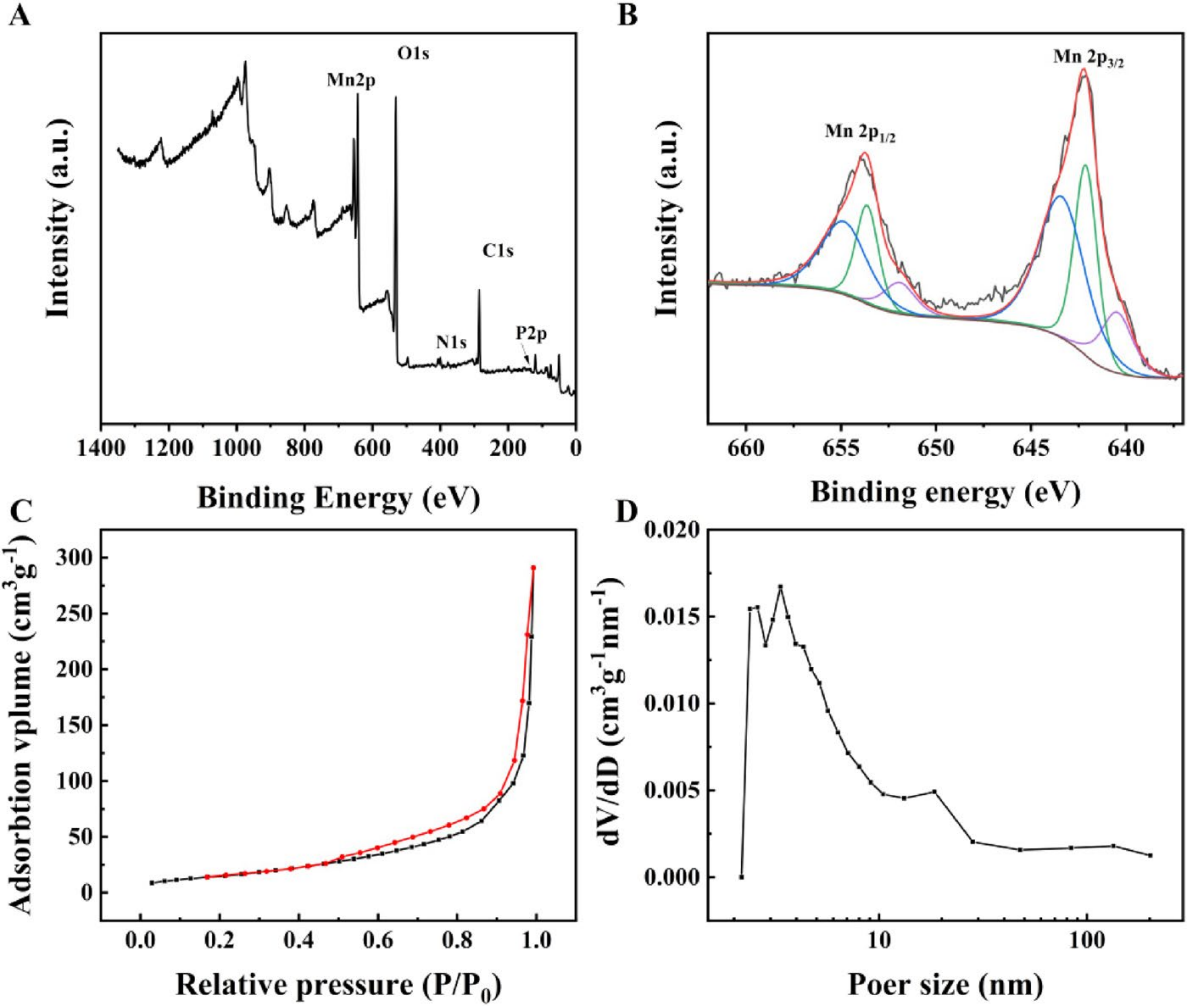
(A) XPS spectra of H-MnO_2_-SRF-APT. (B) Detailed XPS spectrum of the Mn element in H-MnO_2_-SRF-APT. (C) Nitrogen absorption–desorption isotherm and (D) pore distribution of H-MnO_2_.

The total XPS spectra of H-MnO_2_-SRF-APT (Figure 4(A)) simultaneously show the signals of O, Mn, N, P, C, and other elements. The detailed XPS spectrum of the Mn element in H-MnO_2_-SRF-APT is shown in Figure 4(B). The 2p orbital electron binding energy of manganese atoms in nanoparticles was selected to be analyzed. The electron binding energy of Mn 2p_3/2_ is 643.39, 642.12, and 640.48 eV respectively, indexing to Mn^4+^, Mn^3+^, and Mn^2+^ species (Zhou et al., 2022). The nitrogen adsorption–desorption isotherm of H-MnO_2_ was collected, and the corresponding specific surface area was calculated to be 58 m^2^ g^−1^ (Figure 4(C)). In addition, this adsorption–desorption isotherm belongs to the typical V-type isotherm, confirming its rich mesopores. The corresponding pore size distribution curve was obtained by BJH model fitting, and the results showed that the average pore size of H-MnO_2_ was 3.6 nm (Figure 4(D)).

**Figure 5.**
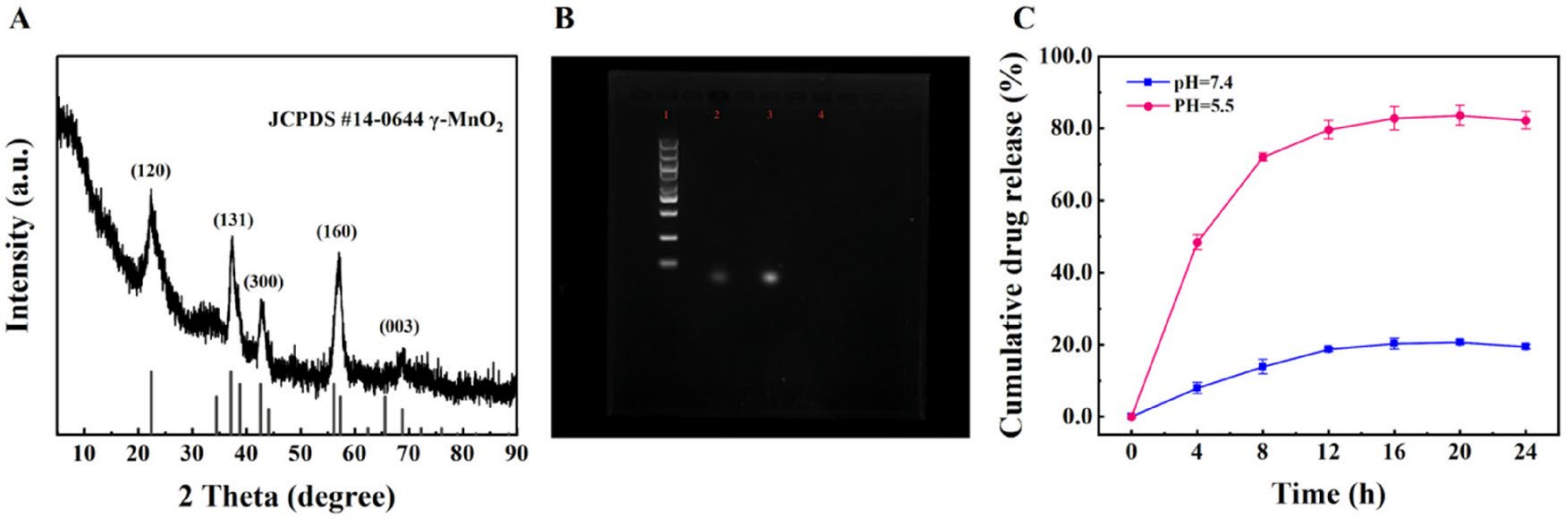
(A) XRD pattern of H-MnO_2_**_._** (B) Nucleic acid image Lane 1 represents Marker; Lane 2 stands for H-MnO_2_-SRF-APT; Lane 3 represents aptamer AP613-1; Lane 4 (no color is displayed) represents H-MnO_2_-SRF demonstrate the ligation of the aptamer. (C) SRF in vitro release curve of H-MnO_2_-SRF-APT at different pH values (pH 5.5 and pH 7.4).

According to the XRD statistics (Figure 5(A)), the characteristic diffraction peaks of H-MnO_2_ at 2*θ* = 23.6°, 37.0°, 42.6°, 56.8°, and 67.6° corresponded to that of the γ-MnO_2_ crystal type (JCPDS 14-0644) appeared at (120), (131), (300), (160), (003), respectively, proved that the obtained H-MnO_2_ has a cubic crystal structure. To further verify if AP613-1 was successfully coupled to the surface of H-MnO_2_-SRF, the evaluation of agarose gel electrophoresis was performed. As shown in Figure 5(B), these results indicated that the H-MnO_2_-SRF-APT nanoprobe was stained with nucleic acid pigments, proving that it has been successfully ligated to the nucleic acid aptamer.

### 3.2. Amounts of loading drugs, *in vitro* drug release

After H-MnO_2_ was stirred with free SRF overnight, the content of loading drugs and the encapsulation rate were determined to be 22% and 88% respectively. Then, the drug release performance of H-MnO_2_-SRF-APT was evaluated at different pH values. As shown in Figure 5(C), the SRF release rate at pH 7.4 was highly slower than that at pH 5.5, in which the rate of the release of SRF at pH 5.5 was as high as 75% after 8 h, while that of SRF at pH 7.4 was only 18%. After 24 h, nearly 90% of the SRF was diffused from H-MnO_2_-SRF-APT under acidic conditions, but only 18% under neutral conditions. This significant pH-dependent drug release behavior could reduce the release of SRF in a neutral environment, prolonging blood circulation in the body, and facilitating SRF to unload free drugs in the TME, thereby reducing adverse side effects on normal tissues.

### 3.3. Cellar uptake experimental results

**Figure 6.**
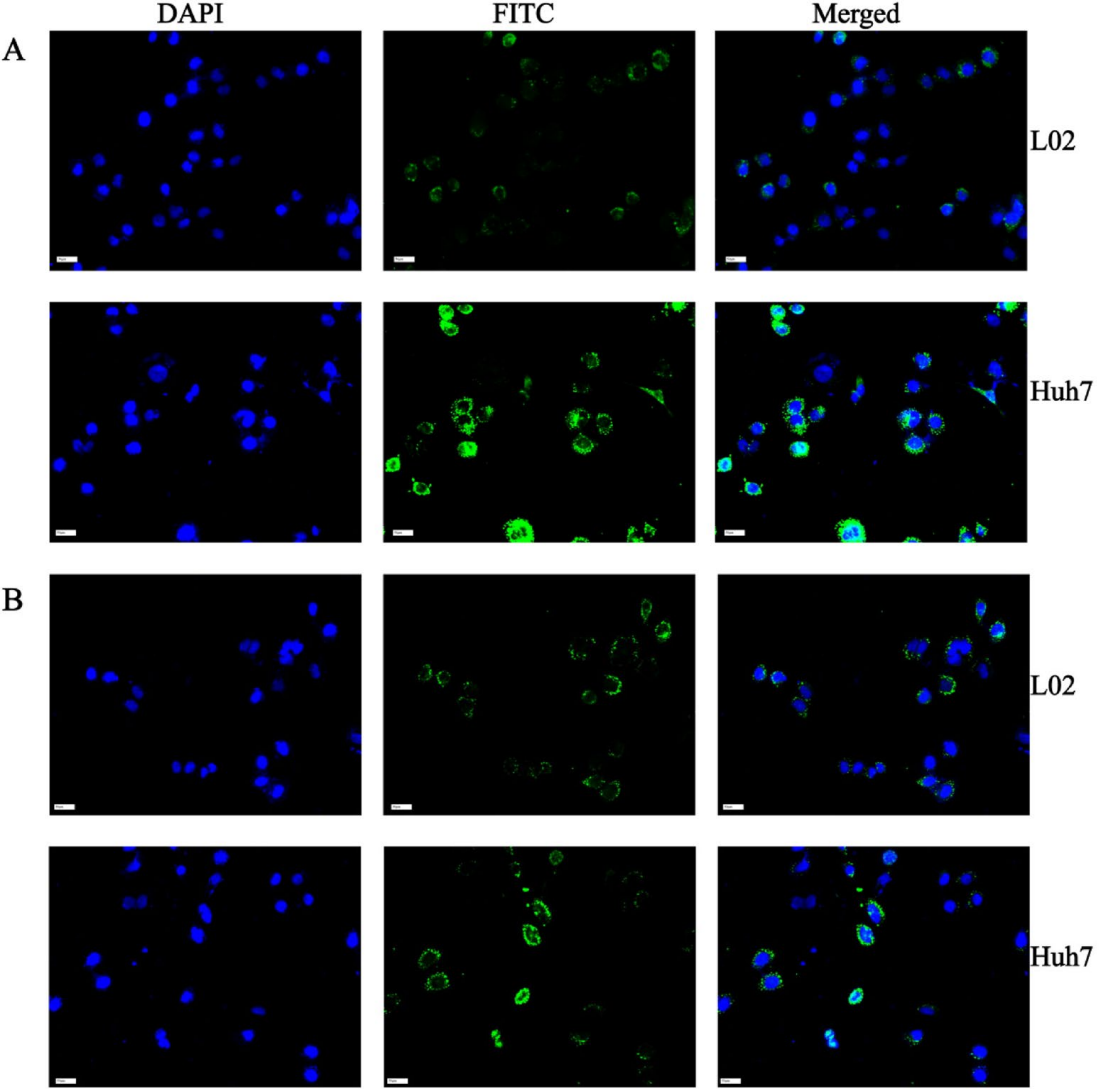
(A) CLSM images of Huh7 and L02 cells after incubation with H-MnO_2_-SRF-APT-FITC and (B) H-MnO_2_-SRF-FITC: blue represents DAPI; green represents FITC-modified nanoparticles.

**Figure 7.**
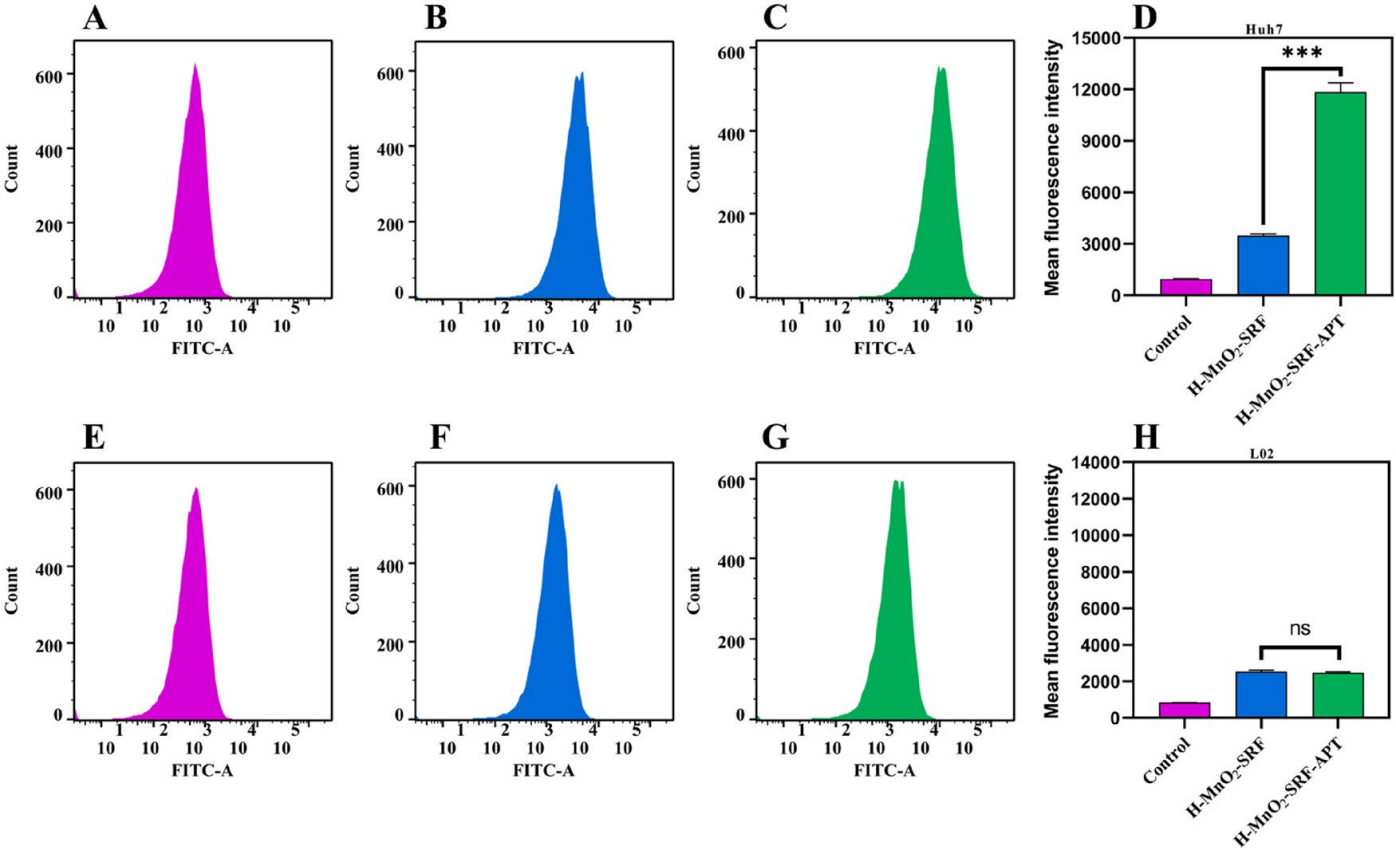
(A–C) Fluorescence of green FITC in Huh7 cells detected by flow cytometry. (D) Mean fluorescence intensity of Huh7 cells treated with PBS, H-MnO_2_-SRF-FITC, and H-MnO_2_-SRF-APT-FITC. (E–G) Fluorescence of green FITC in L02 cells detected by flow cytometry. (H) Mean fluorescence intensity of L02 cells treated with PBS, H-MnO_2_-SRF-FITC, and H-MnO_2_-SRF-APT-FITC.

The targeted receptor GPC3 will bind to aptamers on the surface of H-MnO_2_-SRF nanoparticles to trigger the release of SRF into tumor cells, exerting a therapeutic effect. GPC3 is reported to be highly expressed in Huh7 (HCC cells) but dormant in L02 (normal hepatocytes). It has been recognized as a biomarker that distinguishes normal liver cells from liver cancer cells (Ho & Kim, 2011). Therefore, using the unmodified targeting nanoparticle H-MnO_2_-SRF-FITC as the control group, the uptake of nanoparticles by cells was studied utilizing these two cell lines. Huh7 and L02 cells were co-cultured with H-MnO_2_-SRF-APT-FITC and H-MnO_2_-SRF-FITC for 8 h, respectively, and the cells are observed and analyzed by CLSM. As shown in Figure 6(A), the uptake capacity of Huh7 cells for H-MnO_2_-SRF-APT-FITC was remarkably higher than that of L02 cells. Besides, there was notably enhanced green fluorescence from Huh7 cells but relatively pale fluorescent emission of L02 cells. For cells treated with H-MnO_2_-SRF-FITC, the green fluorescence from Huh7 and L02 cells was both bright with little difference (Figure 6(B)). To further investigate the uptake of H-MnO_2_-SRF-APT in GPC3-positive expressing Huh7 cells, we performed fluorescence quantification by flow cytometry. As shown in Figure 7(A–C), the mean fluorescence intensity of the FITC-H-MnO_2_-SRF-APT incubation group was significantly higher than that of the H-MnO_2_-SRF group, while there was little difference in the mean fluorescence intensity in the two experimental groups of L02 cells. The aforementioned results demonstrated that H-MnO_2_-SRF-APT had the ability of specific recognition for tumors.

### 3.4. Cytotoxicity and proliferation inhibition experiments

**Figure 8.**
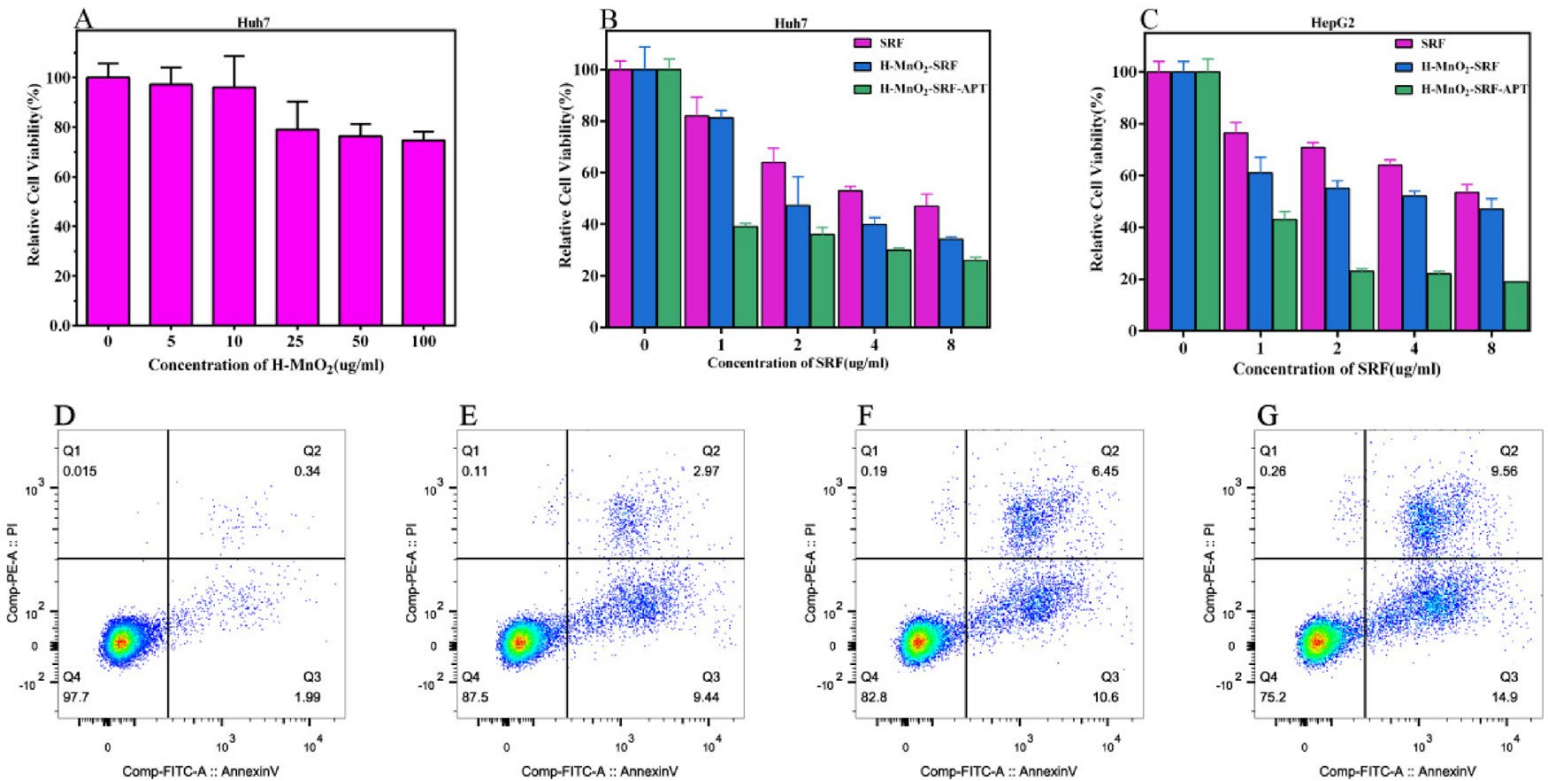
(A) Relative viability of Huh7 cells after incubation with different concentrations of H-MnO_2_. (B and C) Relative viability of Huh7 and HepG2 cells after incubation with free SRF, H-MnO_2_-SRF and H-MnO_2_-SRF-APT at different SRF concentrations. (D–G) The apoptosis rates of Huh7 cells co-cultured with PBS, free SRF, H-MnO_2_-SRF, and H-MnO_2_-SRF-APT nanoparticles for flow cytometry apoptosis data.

The biosecurity of drug carriers is critical for further applications. As shown in Figure 8(A), the cell survival rate of Huh7 is still above 74% at the high concentration of H-MnO_2_ nanoparticles (up to 100 ug/mL), indicating the good biocompatibility of the carrier.

Subsequently, as shown in Figure 8(B,C), the therapeutic effect of H-MnO_2_-SRF-APT was evaluated using HCC Huh7 and HepG2 cells. Obviously, with the increased concentration of SRF, the mortality of Huh7, as well as HepG2 cells in the H-MnO_2_-SRF-APT group, increased significantly compared with that in the free SRF group. More importantly, at the equivalent concentrations of SRF, the mortality of the H-MnO_2_-SRF-APT group was significantly higher than that of the non-targeted H-MnO_2_-SRF group, indicating that H-MnO_2_-SRF-APT has a better ability to inhibit tumor proliferation and could kill liver cancer cells in a targeted way.

### 3.5. Apoptosis experimental results

The results of flow cytometry are shown in Figure 8(D–G). The apoptotic rates of Huh7 cells treated with PBS, free SRF, H-MnO_2_-SRF, and H-MnO_2_-SRF-APT are 2.33%, 12.41%, 17.05%, and 24.46%, respectively. Compared to free SRF, H-MnO_2_ nanoparticle-loaded SRF could induce more apoptosis of Huh7 cells. On the other hand, H-MnO_2_-SRF-APT resulted in an 8% higher apoptosis rate in Huh7 cells than that of H-MnO_2_-SRF. Accordingly, the successful connection between targeting substance APT and nanoparticles could improve the targeting of nanoparticles, and effectively enhance the apoptosis of liver cancer cells, which strengthens the therapeutic effect of SRF.

### 3.6. Magnetic resonance imaging experimental results

**Figure 9.**
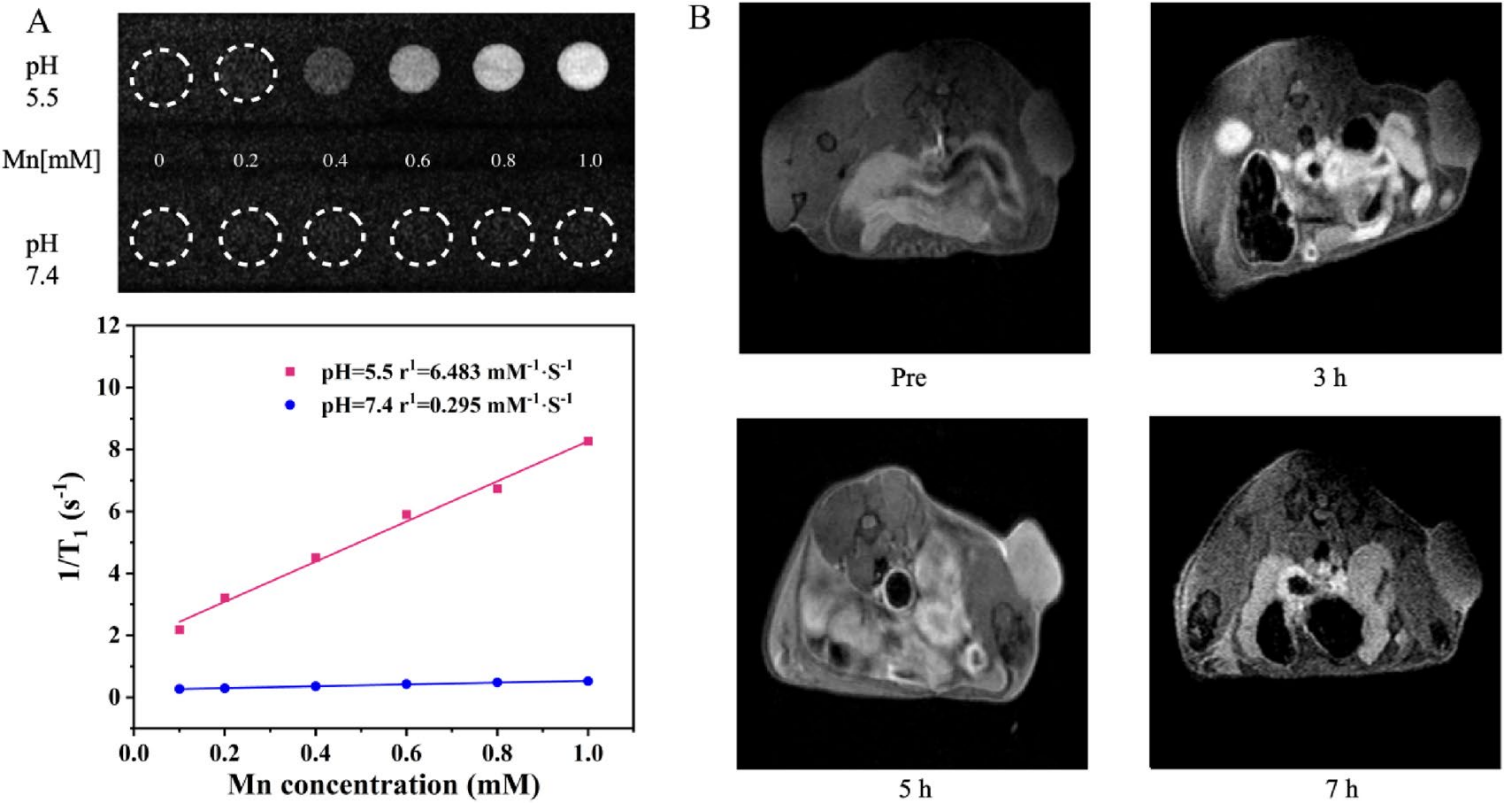
(A) T_1_-weighted MR images and the transverse relativities (*r*_1_) of H-MnO_2_-SRF-APT dispersion at different pH conditions. (B) T_1_-weighted MRI images of Huh7 tumor-bearing nude mice before and after tail vein injection (3 h, 5 h, and 7 h) of H-MnO_2_-SRF-APT.

To verify the MRI capability of the nanoparticles, MR T_1_ imaging was performed after soaking for 12 h under different pH (5.5 and 7.4) according to the Mn^2+^ content (0–1 mM) of H-MnO_2_-SRF-APT. As shown in Figure 9(A), the T_1_-weighted MR signal was enhanced under acidic conditions (pH = 5.5) by increasing the concentration of Mn^2+^ ions, in which its relaxation rate was calculated to be 6.48 mM^−1^ S^–1^. However, increasing the concentration of Mn^2+^ ion seems to have no effect on the signal intensity whose relaxation rate was calculated to be only 0.295 mM^−1^ S^–1^. The total results indicated that H-MnO_2_-SRF-APT is relatively stable under neutral conditions but degraded to produce Mn^2+^ ions under acidic conditions, thereby achieving the T_1_ image.

The in vivo imaging of H-MnO_2_-SRF-APT is shown in Figure 9(B). After the injection of nanoparticles, the signal of the T_1_ image within the tumor increases gradually over time, reaching its summit at 5 h. Therefore, H-MnO_2_-SRF-APT could be used as an acid-responsive T_1_ contrast agent.

### 3.7. *In vivo* tumor treatment and toxicity assessments

**Figure 10.**
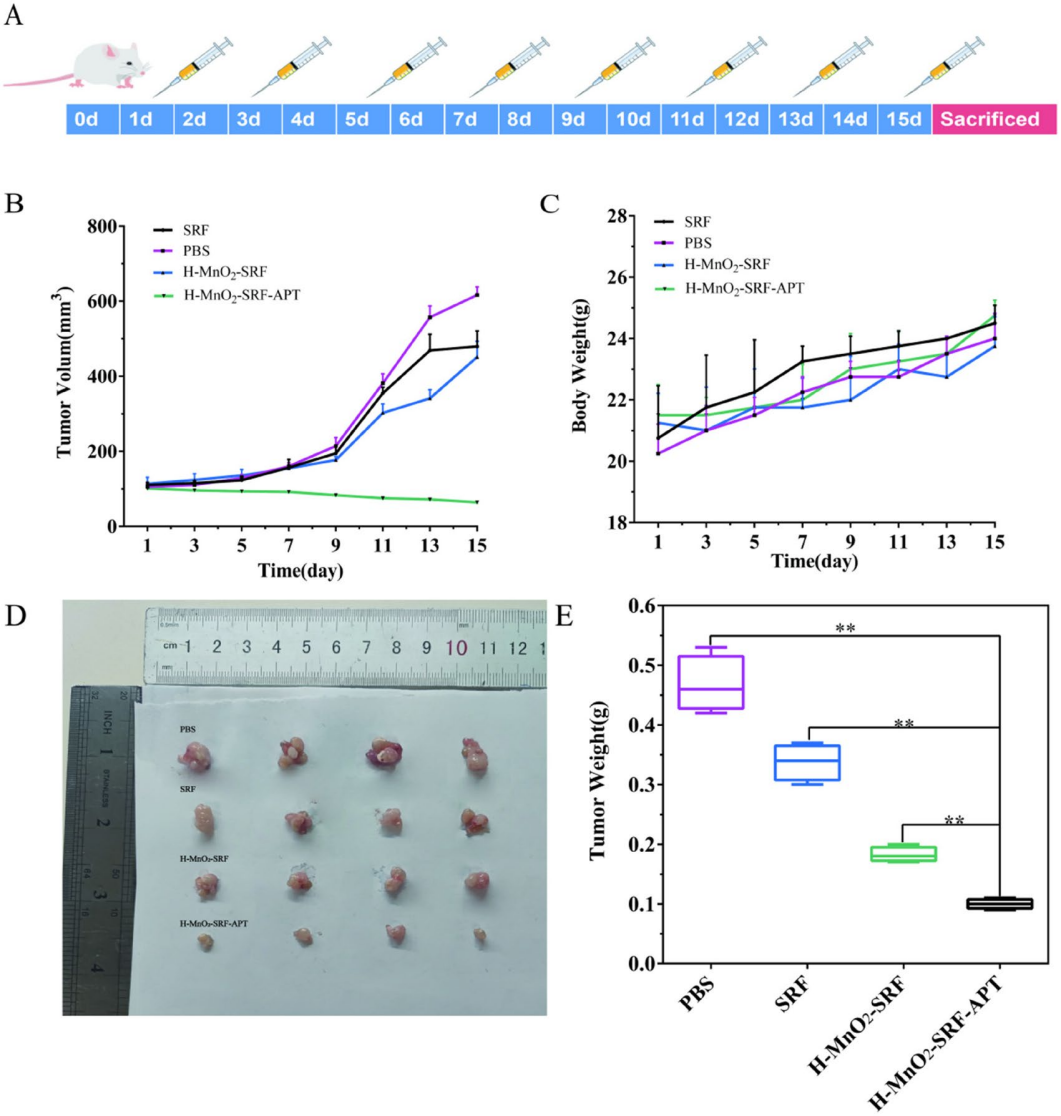
(A) Schematic diagram of treatment with PBS, free SRF, H-MnO_2_-SRF, and H-MnO_2_-SRF-APT in BALB-C nude mouse tumor model. (B) Tumor volume growth trend, (C) body weight of nude mice in different treatment groups, (D) tumor treatment effect graph, and (E) final mean tumor weight after 15 days of treatment.

As shown in Figure 10(A), the tumor-bearing nude mice were grouped for the treatment for 15 days. Figure 10(B) shows that no obvious inhibition was observed on the tumor volume of nude mice in the PBS group, but the tumor growth rates of the free SRF group and the H-MnO_2_-SRF group were inhibited to a certain extent after treatment for 15 days. Besides, the tumor volume of the naked mice in the H-MnO_2_-SRF-APT group was significantly reduced. At the same time, as shown in Figure 10(C), there was no significant difference in body weight in each group of mice. At the end of the experiment, the mice were sacrificed, and the tumors were taken out to take photos and weight. As shown in Figure 10(D), the H-MnO_2_-SRF-APT group had the best therapeutic effect.

The final tumor weight is shown in Figure 10(E). The mean tumor weights in the PBS, free SRF, H-MnO_2_-SRF, and H-MnO_2_-SRF-APT groups were 0.467 g, 0.337 g, 0.182 g, and 0.100 g, respectively, which are consistent with the growing trend of tumor volume. These results further confirmed that H-MnO_2_-SRF-APT exhibited a more significant therapeutic effect than free SRF.

**Figure 11.**
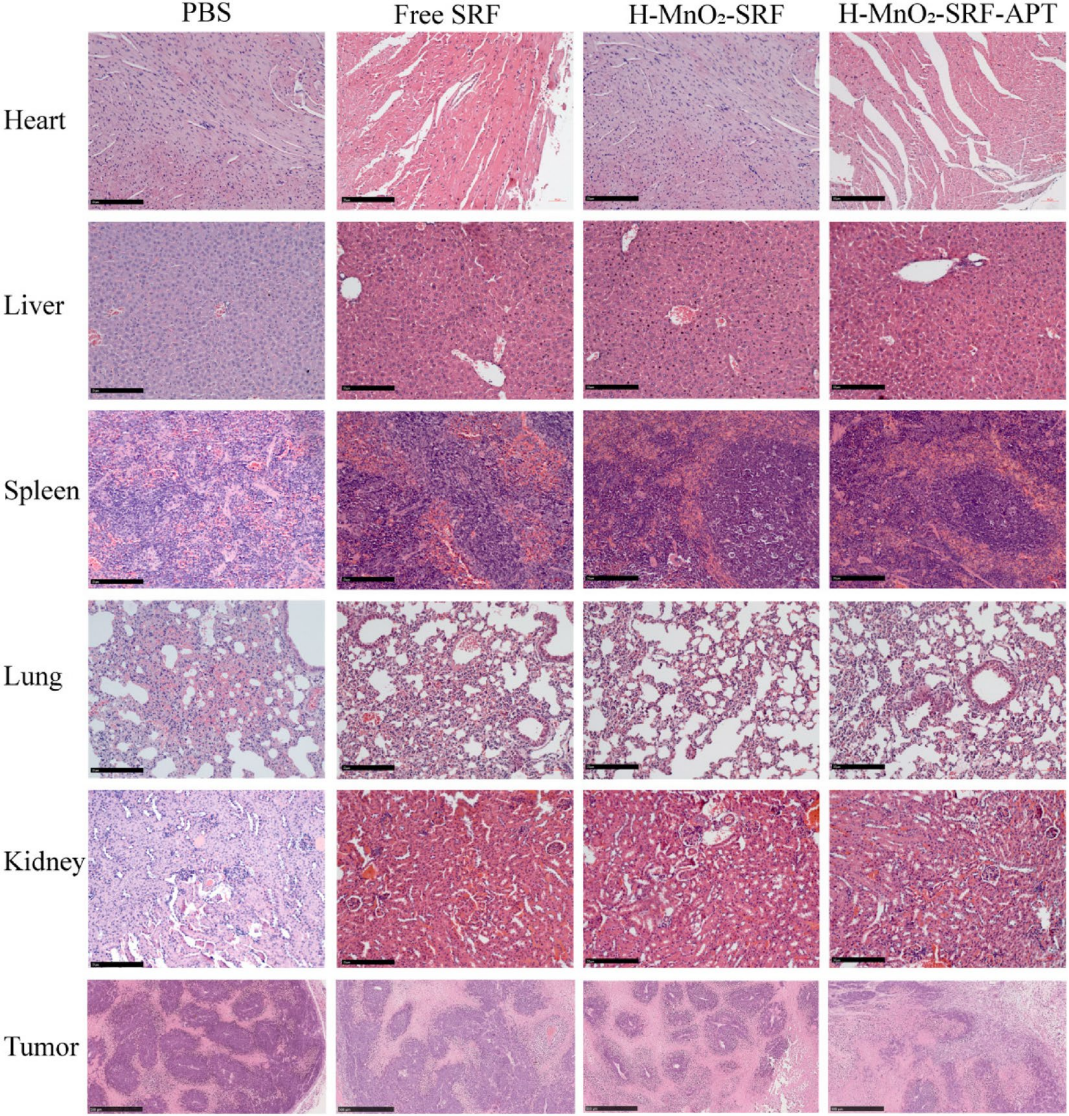
H&E staining of heart, liver, spleen, lung, kidney and tumor sections collected from mice in each group after the process (scale: 50 μm).

In addition, as shown in Figure 11, no significant pathological changes were observed in the organs as analyzing the histopathological changes in major organs (heart, liver, spleen, lungs, and kidneys) proving that H-MnO_2_-SRF-APT and H-MnO_2_-SRF did not cause evident damage to major organs.

There was no significant necrosis of tumors in the PBS group, but the free SRF group, H-MnO_2_-SRF, and H-MnO_2_-SRF-APT group all showed that the interstitial area was significantly increased and the significantly increased infiltrating inflammatory cells in the stroma densely were distributed around the cancer nest. Specifically, compared with the PBS group and the free SRF group, the number of cancer nests and cancer cells in the H-MnO_2_-SRF-APT group were significantly reduced, and the size of the cancer cell nest became smaller or almost disappeared. It shows that cancer cells are very sensitive to H-MnO_2_-SRF-APT, further indicating that H-MnO_2_-SRF-APT can improve the effect of SRF in the treatment of liver cancer.

## 4. Conclusion

In this study, H-MnO_2_-SRF-APT with pH response function was successfully developed to improve the efficacy of SRF in the treatment of HCC. The pH-responsive degradation of H-MnO_2_ allows for the efficient release of SRF while enabling tumor-specific imaging. The target recognition ability of H-MnO_2_-SRF-APT nanoparticles can be intuitively observed by CLSM, effectively increasing the uptake of SRF by HCC cells and reducing side effects. Finally, both *in vitro* and *in vivo* experimental results proved that H-MnO_2_-SRF-APT could more effectively inhibit the growth of HCC cells and have good biosecurity. Therefore, H-MnO_2_-SRF-APT has great application potential in the diagnosis and treatment of tumors, providing a new strategy for cancer diagnosis and treatment.

## Data Availability

The data and materials in the current study are available from the first author upon reasonable request.
